# Di-μ-chlorido-chlorido-1κ*Cl*-(μ-1-phenyl-3-phenyl­imino­but-1-enolato-1:2κ^3^ *O*:*O*,*N*)bis­(1-phenyl-3-phenyl­imino­but-1-enolato)-1κ^2^ *N*,*O*;2κ^2^ *N*,*O*-dichromium(III) toluene disolvate

**DOI:** 10.1107/S1600536808027645

**Published:** 2008-09-06

**Authors:** Liming Tang, Zuo-Xi Li

**Affiliations:** a State Key Laboratory of Polymer Chemistry and Physics, Changchun Institute of Applied Chemistry, Chinese Academy of Sciences, No. 5625 Renmin Street, Changchun 130022, People’s Republic of China; b Department of Chemistry, Nankai University, Tianjin 300071, People’s Republic of China

## Abstract

In the title dichromium complex, [Cr_2_(C_16_H_14_NO)_3_Cl_3_]·2C_7_H_8_, each Cr^III^ atom has a distorted octa­hedral coordination geometry. The complex mol­ecule has three six-membered chelate rings formed by hydroxy­butane­imine ligands and the two Cr^III^ atoms are bridged by two Cl atoms and one O atom.

## Related literature

For related literature, see: Abbati (2005 ▶); Ballem *et al.* (2004 ▶); Cole & Gibson (1994 ▶); Gibson *et al.* (2000 ▶); Jones *et al.* (2005 ▶); Karol *et al.* (1972 ▶); MacAdams *et al.* (2005 ▶); Smith (2005 ▶); Theopold (1998 ▶).
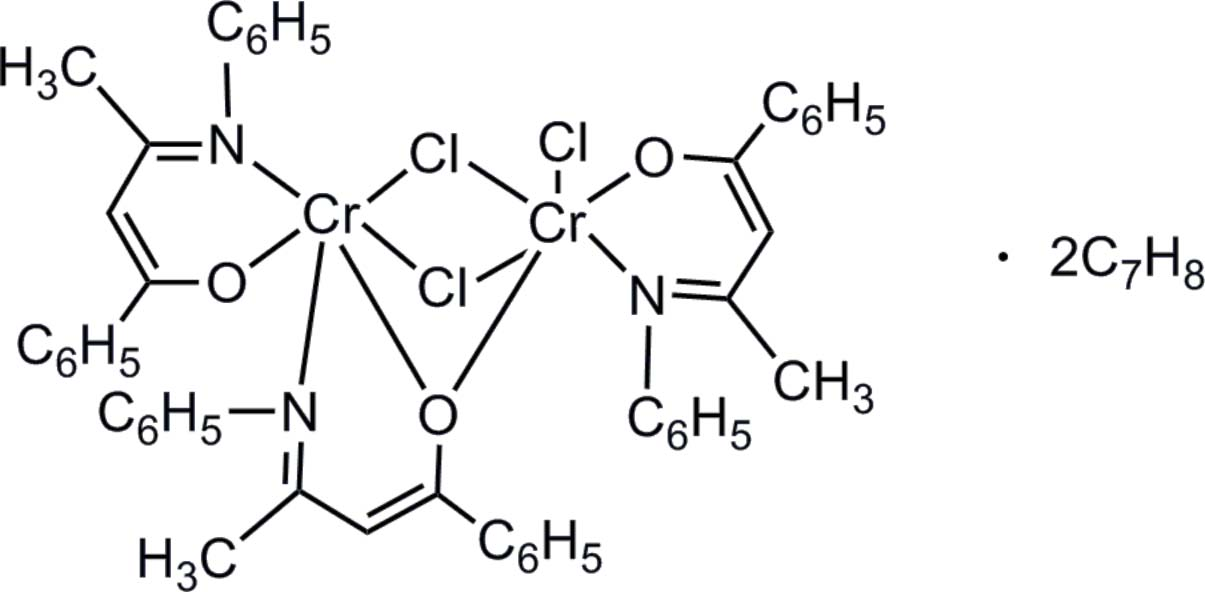


## Experimental

### 

#### Crystal data

[Cr_2_(C_16_H_14_NO)_3_Cl_3_]·2(C_7_H_8_)
*M* *_r_* = 1103.46
Monoclinic, 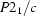

*a* = 17.829 (4) Å
*b* = 12.644 (3) Å
*c* = 25.038 (5) Å
β = 90.90 (3)°
*V* = 5643 (2) Å^3^
*Z* = 4
Mo *K*α radiation
μ = 0.57 mm^−1^
*T* = 293 (2) K
0.45 × 0.42 × 0.08 mm

#### Data collection

Bruker SMART APEX CCD diffractometer
Absorption correction: multi-scan (**SADABS**; Sheldrick, 1996 ▶) *T*_min_ = 0.772, *T*_max_ = 0.955
28782 measured reflections
9904 independent reflections
7777 reflections with *I* > 2σ(*I*)
*R*_int_ = 0.024

#### Refinement

*R*[*F* ^2^ > 2σ(*F* ^2^)] = 0.048
*wR*(*F* ^2^) = 0.153
*S* = 1.02
9904 reflections
611 parameters
H-atom parameters constrained
Δρ_max_ = 0.88 e Å^−3^
Δρ_min_ = −0.50 e Å^−3^

### 

Data collection: *SMART* (Bruker, 1998 ▶); cell refinement: *SAINT-NT* (Bruker, 2003 ▶); data reduction: *SAINT-NT*; program(s) used to solve structure: *SHELXS97* (Sheldrick, 2008 ▶); program(s) used to refine structure: *SHELXL97* (Sheldrick, 2008 ▶); molecular graphics: *SHELXTL* (Sheldrick, 2008 ▶); software used to prepare material for publication: *SHELXTL*.

## Supplementary Material

Crystal structure: contains datablocks I, global. DOI: 10.1107/S1600536808027645/is2322sup1.cif

Structure factors: contains datablocks I. DOI: 10.1107/S1600536808027645/is2322Isup2.hkl

Additional supplementary materials:  crystallographic information; 3D view; checkCIF report

## Figures and Tables

**Fig. 1.**
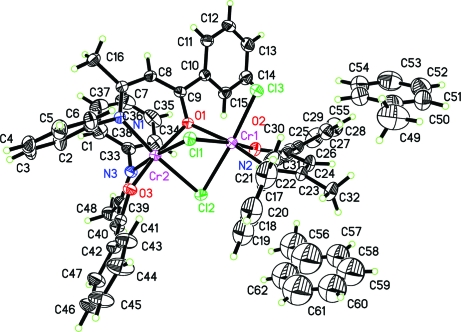
The molecular structure of title compound with atom labels. Displacement ellipsoids are shown at the 50% probability level. H atoms not involved in hydrogen bonding have been omitted for clarity.

## supplementary crystallographic information

### Comment

In recent years organochromium complexes have received more and more attentions as heterogenous or homogeneous catalysts for olefin polymerization (MacAdams *et al.*, 2005; Smith 2005; Jones *et al.*, 2005). Previously, much research effort has been dedicated in the synthesis of half-sandwich chromium complexes as model systems for the unipol family of silica-supported chromium catalysts which can produce high density polyethylene (Theopold *et al.*, 1998; Karol *et al.*, 1972; Cole & Gibson, 1994). Recently, a number of non-cyclopentadienyl ligand have been investigated with a view to synthesizing high active, single site or well define olefin polymerizaton catalysts, which have provided new opportunities for the production of polymers with tailored structures and novel properties. Hence, our work aimed to synthesize the new chromium complex with β-diketiminato ligand. Here, we report the synthesis and structure of the first such species, [(Ph)NC(Ph)C(H)C(CH_3_)OCr]_2_(µ-Cl)_2_[µ-O(CH_3_)CC(H)(Ph)CN(Ph)] Cl.2C_7_H_8_.

The title complex crystallized in the monoclinic system and the structure consists of three six-membered chelate rings, as illustrated in Figure 1. Two unsymmetric chromium centres are in a distorted octahedral environment held together by one oxygen and two chloride bridges. The two Cr atoms are in distinct stereochemical environment. Atom Cr1 is coordinated by two β-diketiminato ligands, two bridging C1 (Cl_b_) atoms and one bridging O atom (O_b_); atom Cr2 is coordinated by one β-diketiminato ligand, one Cl atom, two Cl_b_ atoms and one O_b_ atom. In each chelate ring the bite angle is near optimal, the respective O—Cr—N angles being in the range 88.80 (10) to 99.22 (10)°.

The range of the Cr—O, Cr—N and Cr—Cl bonds lengths (Table 1) are 1.890–2.138, 2.016–2.019 and 2.3954–2.4145 Å, respectively. These are comparable to the bond lengths for the reported mononuclear chromium complexes with salicylaldiminato ligands (Gibson *et al.*, 2000), which have Cr—O bond length of 1.947 (3) Å, Cr—N bond length of 2.074 (4) Å and Cr—Cl bond length of 2.320 (1) Å. In addition, the Cr—Cl distance for Cr1 atom bonded to one terminate Cl is somewhat shorter than those for Cr2 bonded to two Cl_b_. The Cr—O distances for atoms bonded to O_b_ are longer than those for Cr bonded to β-diketiminato ligand.

### Experimental

Reaction of CrCl_3_(THF)_3_ with equivalent lithium salt of β-diketiminate in dried tetrahydrofuran for 20 h at ambient temperature followed by evaporation of the solvent in vacuum to yield a crude product. To the crude product was added dried CH_2_Cl_2_, and the mixture was stirred for 10 min and then filtered. The filtrate was evaporated in vacuum to afford a solid residue, which was dissolved by toluene and layered with n-hexane. The brown crystals of the title compound were obtained in 62% yield.

### Refinement

H atoms were positioned geometrically (C—H = 0.93–0.96 Å) and refined in the riding-model approximation, with *U*_iso_(H) = 1.2*U*_eq_(C) or 1.5*U*_eq_(methyl C). For one of the toluene molecules, atoms C56–C62 were refined isotropically to avoid abnormal deformation of the displacement ellipsoids.

### Crystal data

**Table:**

[Cr_2_(C_16_H_14_NO)_3_Cl_3_]·2(C_7_H_8_)	*F*(000) = 2296
*M**_r_* = 1103.46	*D*_x_ = 1.299 Mg m^−^^3^
Monoclinic, *P*2_1_/*c*	Mo *K*α radiation, λ = 0.71073 Å
Hall symbol: -P 2ybc	Cell parameters from 5147 reflections
*a* = 17.829 (4) Å	θ = 2.3–26.0°
*b* = 12.644 (3) Å	µ = 0.57 mm^−^^1^
*c* = 25.038 (5) Å	*T* = 293 K
β = 90.90 (3)°	Block, blue
*V* = 5643 (2) Å^3^	0.45 × 0.42 × 0.08 mm
*Z* = 4	

### Data collection

**Table:**

Bruker SMART APEX CCD diffractometer	9904 independent reflections
Radiation source: fine-focus sealed tube	7777 reflections with *I* > 2σ(*I*)
graphite	*R*_int_ = 0.024
Detector resolution: 8 pixels mm^-1^	θ_max_ = 25.0°, θ_min_ = 1.6°
φ and ω scans	*h* = −21→19
Absorption correction: multi-scan (*SADABS*; Sheldrick, 1996)	*k* = −14→14
*T*_min_ = 0.772, *T*_max_ = 0.955	*l* = −29→27
28782 measured reflections	

### Refinement

**Table:**

Refinement on *F*^2^	Primary atom site location: structure-invariant direct methods
Least-squares matrix: full	Secondary atom site location: difference Fourier map
*R*[*F*^2^ > 2σ(*F*^2^)] = 0.048	Hydrogen site location: inferred from neighbouring sites
*wR*(*F*^2^) = 0.153	H-atom parameters constrained
*S* = 1.02	*w* = 1/[σ^2^(*F*_o_^2^) + (0.0908*P*)^2^ + 4.1962*P*] where *P* = (*F*_o_^2^ + 2*F*_c_^2^)/3
9904 reflections	(Δ/σ)_max_ = 0.014
611 parameters	Δρ_max_ = 0.88 e Å^−^^3^
0 restraints	Δρ_min_ = −0.50 e Å^−^^3^

### Special details

**Table:**

Geometry. All e.s.d.'s (except the e.s.d. in the dihedral angle between two l.s. planes) are estimated using the full covariance matrix. The cell e.s.d.'s are taken into account individually in the estimation of e.s.d.'s in distances, angles and torsion angles; correlations between e.s.d.'s in cell parameters are only used when they are defined by crystal symmetry. An approximate (isotropic) treatment of cell e.s.d.'s is used for estimating e.s.d.'s involving l.s. planes.
Refinement. Refinement of *F*^2^ against ALL reflections. The weighted *R*-factor *wR* and goodness of fit *S* are based on *F*^2^, conventional *R*-factors *R* are based on *F*, with *F* set to zero for negative *F*^2^. The threshold expression of *F*^2^ > σ(*F*^2^) is used only for calculating *R*-factors(gt) *etc*. and is not relevant to the choice of reflections for refinement. *R*-factors based on *F*^2^ are statistically about twice as large as those based on *F*, and *R*- factors based on ALL data will be even larger.

### Fractional atomic coordinates and isotropic or equivalent isotropic displacement parameters (Å^2^)

**Table:**

	*x*	*y*	*z*	*U*_iso_*/*U*_eq_	
Cr1	0.21300 (3)	0.47166 (4)	0.408402 (18)	0.03352 (14)	
Cr2	0.27644 (3)	0.57522 (4)	0.506694 (18)	0.03328 (14)	
Cl1	0.21103 (4)	0.65638 (6)	0.43236 (3)	0.04033 (19)	
Cl2	0.34183 (4)	0.47921 (6)	0.43935 (3)	0.04182 (19)	
Cl3	0.08908 (4)	0.46873 (7)	0.38655 (3)	0.0484 (2)	
C1	0.23621 (17)	0.7543 (3)	0.57680 (12)	0.0412 (7)	
C2	0.2849 (2)	0.7659 (3)	0.61958 (16)	0.0660 (11)	
H2A	0.3020	0.7067	0.6381	0.079*	
C3	0.3084 (3)	0.8663 (4)	0.63509 (18)	0.0749 (12)	
H3A	0.3405	0.8736	0.6645	0.090*	
C4	0.2857 (2)	0.9530 (3)	0.60827 (17)	0.0663 (11)	
H4A	0.3016	1.0198	0.6192	0.080*	
C5	0.2388 (3)	0.9422 (3)	0.56473 (17)	0.0680 (11)	
H5A	0.2233	1.0019	0.5458	0.082*	
C6	0.2142 (2)	0.8428 (3)	0.54864 (15)	0.0582 (9)	
H6A	0.1829	0.8360	0.5188	0.070*	
C7	0.14930 (17)	0.6124 (2)	0.57870 (12)	0.0397 (7)	
C8	0.11773 (17)	0.5134 (2)	0.56223 (12)	0.0401 (7)	
H8A	0.0768	0.4908	0.5818	0.048*	
C9	0.13845 (16)	0.4473 (2)	0.52229 (11)	0.0345 (6)	
C10	0.09372 (16)	0.3508 (2)	0.51082 (11)	0.0362 (6)	
C11	0.01696 (17)	0.3492 (3)	0.52023 (13)	0.0436 (7)	
H11A	−0.0066	0.4097	0.5329	0.052*	
C12	−0.02463 (19)	0.2581 (3)	0.51088 (15)	0.0533 (9)	
H12A	−0.0758	0.2578	0.5174	0.064*	
C13	0.0094 (2)	0.1683 (3)	0.49198 (15)	0.0581 (9)	
H13A	−0.0186	0.1073	0.4859	0.070*	
C14	0.0855 (2)	0.1689 (3)	0.48209 (15)	0.0545 (9)	
H14A	0.1086	0.1085	0.4690	0.065*	
C15	0.12709 (19)	0.2591 (2)	0.49162 (13)	0.0447 (7)	
H15A	0.1783	0.2587	0.4851	0.054*	
C16	0.1053 (2)	0.6699 (3)	0.62084 (15)	0.0586 (10)	
H16A	0.1301	0.7352	0.6297	0.088*	
H16B	0.1024	0.6266	0.6522	0.088*	
H16C	0.0557	0.6844	0.6074	0.088*	
C17	0.2615 (2)	0.5963 (3)	0.31358 (12)	0.0480 (8)	
C18	0.3346 (3)	0.6290 (4)	0.31626 (18)	0.0795 (13)	
H18A	0.3718	0.5847	0.3300	0.095*	
C19	0.3523 (4)	0.7303 (4)	0.2979 (2)	0.1081 (13)	
H19A	0.4016	0.7542	0.2990	0.130*	
C20	0.2965 (4)	0.7934 (4)	0.2784 (2)	0.1081 (13)	
H20A	0.3085	0.8611	0.2667	0.130*	
C21	0.2244 (4)	0.7616 (4)	0.2753 (2)	0.1081 (13)	
H21A	0.1876	0.8057	0.2609	0.130*	
C22	0.2064 (3)	0.6625 (3)	0.29382 (16)	0.0691 (11)	
H22A	0.1567	0.6401	0.2930	0.083*	
C23	0.2389 (2)	0.4162 (3)	0.29538 (12)	0.0454 (8)	
C24	0.2233 (2)	0.3093 (3)	0.30801 (13)	0.0531 (9)	
H24A	0.2149	0.2632	0.2796	0.064*	
C25	0.21957 (18)	0.2683 (3)	0.35811 (13)	0.0434 (7)	
C26	0.2115 (2)	0.1529 (3)	0.36700 (14)	0.0489 (8)	
C27	0.1626 (3)	0.0931 (3)	0.33530 (18)	0.0660 (11)	
H27A	0.1351	0.1256	0.3080	0.079*	
C28	0.1548 (3)	−0.0142 (4)	0.3441 (2)	0.0922 (16)	
H28A	0.1216	−0.0537	0.3232	0.111*	
C29	0.1962 (4)	−0.0620 (4)	0.3841 (3)	0.0997 (19)	
H29A	0.1911	−0.1342	0.3901	0.120*	
C30	0.2453 (4)	−0.0041 (4)	0.4152 (2)	0.0882 (16)	
H30A	0.2737	−0.0373	0.4418	0.106*	
C31	0.2525 (3)	0.1036 (3)	0.40705 (16)	0.0644 (10)	
H31A	0.2851	0.1429	0.4286	0.077*	
C32	0.2546 (3)	0.4401 (3)	0.23774 (14)	0.0646 (11)	
H32A	0.2648	0.5143	0.2338	0.097*	
H32B	0.2117	0.4214	0.2161	0.097*	
H32C	0.2973	0.4001	0.2266	0.097*	
C33	0.29759 (18)	0.4312 (3)	0.59879 (13)	0.0461 (8)	
C34	0.2772 (2)	0.3323 (3)	0.58094 (16)	0.0595 (10)	
H34A	0.2939	0.3076	0.5482	0.071*	
C35	0.2313 (3)	0.2693 (4)	0.6126 (2)	0.0780 (13)	
H35A	0.2168	0.2025	0.6007	0.094*	
C36	0.2076 (3)	0.3055 (4)	0.6607 (2)	0.0845 (15)	
H36A	0.1763	0.2635	0.6811	0.101*	
C37	0.2289 (3)	0.4015 (4)	0.67933 (19)	0.0834 (14)	
H37A	0.2131	0.4249	0.7125	0.100*	
C38	0.2750 (2)	0.4654 (3)	0.64824 (15)	0.0637 (10)	
H38A	0.2904	0.5311	0.6610	0.076*	
C39	0.41098 (18)	0.5142 (3)	0.56965 (13)	0.0449 (7)	
C40	0.45142 (18)	0.5924 (3)	0.54261 (14)	0.0484 (8)	
H40A	0.5035	0.5876	0.5438	0.058*	
C41	0.42037 (17)	0.6754 (3)	0.51448 (12)	0.0419 (7)	
C42	0.46498 (18)	0.7627 (3)	0.49090 (14)	0.0482 (8)	
C43	0.4312 (2)	0.8270 (4)	0.45372 (19)	0.0743 (12)	
H43A	0.3815	0.8147	0.4438	0.089*	
C44	0.4693 (3)	0.9097 (4)	0.4307 (2)	0.0987 (18)	
H44A	0.4454	0.9525	0.4055	0.118*	
C45	0.5421 (3)	0.9286 (4)	0.4450 (2)	0.0965 (17)	
H45A	0.5672	0.9863	0.4308	0.116*	
C46	0.5781 (3)	0.8632 (4)	0.4801 (2)	0.0844 (14)	
H46A	0.6286	0.8744	0.4883	0.101*	
C47	0.5405 (2)	0.7801 (4)	0.50358 (17)	0.0681 (11)	
H47A	0.5654	0.7360	0.5277	0.082*	
C48	0.4557 (2)	0.4393 (3)	0.60547 (16)	0.0628 (10)	
H48A	0.4222	0.3898	0.6217	0.094*	
H48B	0.4814	0.4792	0.6328	0.094*	
H48C	0.4916	0.4018	0.5845	0.094*	
C49	0.0647 (5)	0.5239 (7)	0.1550 (4)	0.164 (3)	
H49A	0.0597	0.5198	0.1168	0.246*	
H49B	0.1157	0.5408	0.1645	0.246*	
H49C	0.0321	0.5780	0.1682	0.246*	
C50	0.0444 (3)	0.4211 (5)	0.1787 (3)	0.0977 (17)	
C51	0.0247 (4)	0.3390 (7)	0.1478 (2)	0.112 (2)	
H51A	0.0247	0.3467	0.1108	0.135*	
C52	0.0043 (4)	0.2433 (7)	0.1698 (4)	0.129 (3)	
H52A	−0.0111	0.1880	0.1477	0.154*	
C53	0.0067 (4)	0.2295 (6)	0.2237 (4)	0.125 (2)	
H53A	−0.0065	0.1651	0.2388	0.150*	
C54	0.0288 (3)	0.3123 (6)	0.2550 (2)	0.1031 (18)	
H54A	0.0313	0.3040	0.2919	0.124*	
C55	0.0473 (3)	0.4063 (5)	0.2333 (2)	0.0929 (16)	
H55A	0.0623	0.4618	0.2554	0.111*	
C56	0.4330 (7)	0.1718 (10)	0.3302 (5)	0.223 (5)*	
H56A	0.4570	0.1989	0.3619	0.335*	
H56B	0.4570	0.1072	0.3198	0.335*	
H56C	0.3811	0.1585	0.3370	0.335*	
C57	0.4390 (10)	0.2464 (16)	0.2888 (8)	0.270 (7)*	
C58	0.4135 (5)	0.2281 (7)	0.2416 (4)	0.146 (3)*	
H58A	0.3866	0.1667	0.2340	0.175*	
C59	0.4269 (7)	0.3020 (10)	0.2023 (5)	0.204 (4)*	
H59A	0.4122	0.2838	0.1677	0.245*	
C60	0.4589 (7)	0.3970 (10)	0.2085 (5)	0.202 (4)*	
H60A	0.4621	0.4503	0.1831	0.242*	
C61	0.4871 (8)	0.3998 (12)	0.2620 (6)	0.238 (6)*	
H61A	0.5141	0.4601	0.2714	0.286*	
C62	0.4798 (7)	0.3256 (11)	0.3018 (5)	0.204 (5)*	
H62A	0.5027	0.3325	0.3353	0.245*	
N1	0.21138 (13)	0.6510 (2)	0.55977 (10)	0.0375 (6)	
N2	0.24170 (14)	0.4922 (2)	0.33154 (10)	0.0388 (6)	
N3	0.33750 (14)	0.5026 (2)	0.56454 (10)	0.0404 (6)	
O1	0.19941 (11)	0.46256 (15)	0.49295 (8)	0.0342 (4)	
O2	0.22622 (12)	0.32403 (17)	0.40153 (8)	0.0431 (5)	
O3	0.34883 (11)	0.68544 (17)	0.50624 (8)	0.0418 (5)	

### Atomic displacement parameters (Å^2^)

**Table:**

	*U*^11^	*U*^22^	*U*^33^	*U*^12^	*U*^13^	*U*^23^
Cr1	0.0333 (3)	0.0352 (3)	0.0322 (3)	0.00034 (19)	0.00170 (19)	−0.00150 (19)
Cr2	0.0289 (2)	0.0363 (3)	0.0347 (3)	−0.00178 (19)	0.00146 (18)	−0.00088 (19)
Cl1	0.0445 (4)	0.0358 (4)	0.0407 (4)	0.0034 (3)	−0.0004 (3)	0.0010 (3)
Cl2	0.0317 (4)	0.0513 (5)	0.0426 (4)	0.0041 (3)	0.0033 (3)	−0.0048 (3)
Cl3	0.0361 (4)	0.0611 (5)	0.0481 (5)	−0.0016 (3)	−0.0026 (3)	−0.0037 (4)
C1	0.0397 (17)	0.0437 (18)	0.0404 (17)	−0.0063 (14)	0.0076 (13)	−0.0102 (14)
C2	0.081 (3)	0.058 (2)	0.059 (2)	−0.003 (2)	−0.017 (2)	−0.0103 (19)
C3	0.082 (3)	0.073 (3)	0.069 (3)	−0.013 (2)	−0.018 (2)	−0.026 (2)
C4	0.076 (3)	0.055 (2)	0.068 (3)	−0.023 (2)	0.017 (2)	−0.023 (2)
C5	0.087 (3)	0.047 (2)	0.070 (3)	−0.011 (2)	0.005 (2)	−0.0064 (19)
C6	0.068 (2)	0.049 (2)	0.058 (2)	−0.0079 (18)	−0.0078 (18)	−0.0062 (17)
C7	0.0355 (16)	0.0445 (17)	0.0392 (16)	−0.0005 (13)	0.0038 (13)	−0.0034 (13)
C8	0.0351 (16)	0.0433 (17)	0.0421 (17)	−0.0044 (13)	0.0079 (13)	−0.0024 (13)
C9	0.0310 (15)	0.0357 (15)	0.0367 (15)	−0.0001 (12)	0.0008 (12)	0.0059 (12)
C10	0.0375 (16)	0.0369 (16)	0.0344 (15)	−0.0038 (12)	0.0010 (12)	0.0027 (12)
C11	0.0376 (16)	0.0443 (18)	0.0490 (18)	0.0005 (14)	0.0014 (14)	−0.0017 (14)
C12	0.0365 (17)	0.059 (2)	0.064 (2)	−0.0107 (16)	0.0010 (15)	−0.0046 (18)
C13	0.060 (2)	0.049 (2)	0.065 (2)	−0.0183 (18)	0.0014 (18)	−0.0055 (18)
C14	0.063 (2)	0.0377 (18)	0.063 (2)	−0.0036 (16)	0.0074 (18)	−0.0066 (16)
C15	0.0446 (18)	0.0408 (17)	0.0491 (18)	−0.0009 (14)	0.0087 (14)	0.0005 (14)
C16	0.055 (2)	0.061 (2)	0.061 (2)	−0.0106 (18)	0.0220 (18)	−0.0201 (18)
C17	0.064 (2)	0.0453 (19)	0.0346 (16)	−0.0054 (16)	0.0088 (15)	−0.0008 (14)
C18	0.080 (3)	0.087 (3)	0.072 (3)	−0.031 (3)	0.002 (2)	0.015 (2)
C19	0.170 (4)	0.068 (2)	0.086 (2)	−0.028 (2)	0.015 (2)	0.0124 (16)
C20	0.170 (4)	0.068 (2)	0.086 (2)	−0.028 (2)	0.015 (2)	0.0124 (16)
C21	0.170 (4)	0.068 (2)	0.086 (2)	−0.028 (2)	0.015 (2)	0.0124 (16)
C22	0.096 (3)	0.058 (2)	0.054 (2)	0.014 (2)	0.013 (2)	0.0062 (19)
C23	0.055 (2)	0.0488 (19)	0.0330 (16)	0.0010 (15)	0.0031 (14)	−0.0039 (14)
C24	0.077 (2)	0.0429 (19)	0.0395 (18)	−0.0013 (17)	0.0032 (17)	−0.0075 (15)
C25	0.0463 (18)	0.0396 (17)	0.0445 (18)	0.0027 (14)	0.0041 (14)	−0.0038 (14)
C26	0.060 (2)	0.0368 (17)	0.0500 (19)	0.0048 (15)	0.0112 (16)	−0.0040 (14)
C27	0.080 (3)	0.044 (2)	0.075 (3)	−0.0032 (19)	0.003 (2)	−0.0066 (19)
C28	0.115 (4)	0.050 (3)	0.112 (4)	−0.017 (3)	0.012 (3)	−0.013 (3)
C29	0.152 (6)	0.040 (2)	0.108 (4)	0.002 (3)	0.036 (4)	0.005 (3)
C30	0.127 (4)	0.063 (3)	0.075 (3)	0.038 (3)	0.021 (3)	0.020 (2)
C31	0.083 (3)	0.052 (2)	0.058 (2)	0.017 (2)	0.006 (2)	0.0038 (18)
C32	0.096 (3)	0.059 (2)	0.0390 (19)	−0.001 (2)	0.0133 (19)	−0.0038 (17)
C33	0.0395 (18)	0.051 (2)	0.0470 (18)	0.0001 (14)	−0.0058 (14)	0.0124 (15)
C34	0.063 (2)	0.049 (2)	0.066 (2)	−0.0008 (18)	−0.0135 (19)	0.0114 (18)
C35	0.080 (3)	0.059 (3)	0.094 (4)	−0.018 (2)	−0.025 (3)	0.030 (2)
C36	0.070 (3)	0.095 (4)	0.088 (4)	−0.014 (3)	−0.002 (3)	0.047 (3)
C37	0.091 (3)	0.095 (4)	0.065 (3)	0.002 (3)	0.018 (2)	0.031 (3)
C38	0.073 (3)	0.068 (3)	0.050 (2)	0.001 (2)	0.0061 (19)	0.0129 (18)
C39	0.0404 (18)	0.0509 (19)	0.0432 (17)	0.0038 (15)	−0.0040 (14)	−0.0023 (15)
C40	0.0321 (17)	0.058 (2)	0.055 (2)	−0.0017 (15)	0.0003 (14)	0.0015 (16)
C41	0.0354 (17)	0.0493 (18)	0.0411 (17)	−0.0060 (14)	0.0038 (13)	−0.0084 (14)
C42	0.0377 (17)	0.052 (2)	0.055 (2)	−0.0071 (15)	0.0067 (15)	−0.0041 (16)
C43	0.054 (2)	0.079 (3)	0.090 (3)	−0.011 (2)	0.004 (2)	0.029 (2)
C44	0.070 (3)	0.101 (4)	0.126 (4)	−0.015 (3)	0.007 (3)	0.057 (3)
C45	0.070 (3)	0.092 (4)	0.128 (5)	−0.030 (3)	0.021 (3)	0.034 (3)
C46	0.054 (2)	0.096 (4)	0.104 (4)	−0.032 (2)	0.006 (2)	0.010 (3)
C47	0.050 (2)	0.075 (3)	0.079 (3)	−0.020 (2)	−0.0023 (19)	0.004 (2)
C48	0.047 (2)	0.075 (3)	0.066 (2)	0.0061 (18)	−0.0123 (18)	0.017 (2)
C49	0.140 (7)	0.155 (7)	0.199 (9)	0.009 (6)	0.009 (6)	0.068 (7)
C50	0.074 (3)	0.116 (5)	0.103 (4)	0.008 (3)	0.006 (3)	0.010 (4)
C51	0.096 (4)	0.161 (7)	0.079 (4)	0.017 (4)	−0.008 (3)	−0.030 (4)
C52	0.097 (5)	0.143 (7)	0.146 (7)	−0.007 (5)	−0.007 (5)	−0.054 (6)
C53	0.100 (5)	0.121 (6)	0.154 (7)	−0.011 (4)	0.011 (5)	0.021 (5)
C54	0.088 (4)	0.139 (6)	0.082 (4)	−0.007 (4)	−0.001 (3)	−0.004 (4)
C55	0.080 (3)	0.117 (5)	0.081 (4)	−0.001 (3)	−0.001 (3)	−0.013 (3)
N1	0.0358 (13)	0.0391 (14)	0.0375 (13)	−0.0026 (11)	0.0019 (10)	−0.0053 (11)
N2	0.0412 (14)	0.0399 (14)	0.0354 (13)	0.0011 (11)	0.0043 (11)	0.0020 (11)
N3	0.0364 (14)	0.0446 (15)	0.0401 (14)	−0.0021 (11)	−0.0006 (11)	0.0026 (11)
O1	0.0310 (10)	0.0366 (11)	0.0349 (10)	−0.0027 (8)	0.0024 (8)	−0.0012 (8)
O2	0.0527 (13)	0.0382 (12)	0.0383 (11)	0.0014 (10)	0.0014 (10)	−0.0013 (9)
O3	0.0320 (11)	0.0425 (12)	0.0510 (12)	−0.0053 (9)	0.0011 (9)	−0.0003 (10)

### Geometric parameters (Å, °)

**Table:**

Cr1—O2	1.890 (2)	C29—H29A	0.9300
Cr1—N2	2.016 (2)	C30—C31	1.383 (6)
Cr1—O1	2.138 (2)	C30—H30A	0.9300
Cr1—Cl3	2.2680 (10)	C31—H31A	0.9300
Cr1—Cl1	2.4116 (10)	C32—H32A	0.9600
Cr1—Cl2	2.4145 (10)	C32—H32B	0.9600
Cr2—O3	1.900 (2)	C32—H32C	0.9600
Cr2—O1	2.005 (2)	C33—C34	1.374 (5)
Cr2—N3	2.019 (3)	C33—C38	1.378 (5)
Cr2—N1	2.019 (2)	C33—N3	1.441 (4)
Cr2—Cl2	2.3954 (10)	C34—C35	1.398 (6)
Cr2—Cl1	2.4101 (10)	C34—H34A	0.9300
C1—C2	1.376 (5)	C35—C36	1.363 (7)
C1—C6	1.376 (5)	C35—H35A	0.9300
C1—N1	1.441 (4)	C36—C37	1.353 (7)
C2—C3	1.390 (6)	C36—H36A	0.9300
C2—H2A	0.9300	C37—C38	1.397 (6)
C3—C4	1.345 (6)	C37—H37A	0.9300
C3—H3A	0.9300	C38—H38A	0.9300
C4—C5	1.370 (6)	C39—N3	1.322 (4)
C4—H4A	0.9300	C39—C40	1.404 (5)
C5—C6	1.389 (5)	C39—C48	1.520 (5)
C5—H5A	0.9300	C40—C41	1.375 (5)
C6—H6A	0.9300	C40—H40A	0.9300
C7—N1	1.306 (4)	C41—O3	1.295 (4)
C7—C8	1.431 (4)	C41—C42	1.489 (5)
C7—C16	1.511 (4)	C42—C43	1.369 (5)
C8—C9	1.359 (4)	C42—C47	1.396 (5)
C8—H8A	0.9300	C43—C44	1.378 (6)
C9—O1	1.335 (3)	C43—H43A	0.9300
C9—C10	1.484 (4)	C44—C45	1.362 (7)
C10—C15	1.391 (4)	C44—H44A	0.9300
C10—C11	1.392 (4)	C45—C46	1.360 (7)
C11—C12	1.388 (5)	C45—H45A	0.9300
C11—H11A	0.9300	C46—C47	1.383 (6)
C12—C13	1.376 (5)	C46—H46A	0.9300
C12—H12A	0.9300	C47—H47A	0.9300
C13—C14	1.382 (5)	C48—H48A	0.9600
C13—H13A	0.9300	C48—H48B	0.9600
C14—C15	1.380 (5)	C48—H48C	0.9600
C14—H14A	0.9300	C49—C50	1.478 (9)
C15—H15A	0.9300	C49—H49A	0.9600
C16—H16A	0.9600	C49—H49B	0.9600
C16—H16B	0.9600	C49—H49C	0.9600
C16—H16C	0.9600	C50—C51	1.339 (9)
C17—C18	1.367 (6)	C50—C55	1.378 (8)
C17—C22	1.376 (6)	C51—C52	1.381 (10)
C17—N2	1.437 (4)	C51—H51A	0.9300
C18—C19	1.399 (7)	C52—C53	1.361 (10)
C18—H18A	0.9300	C52—H52A	0.9300
C19—C20	1.360 (9)	C53—C54	1.363 (9)
C19—H19A	0.9300	C53—H53A	0.9300
C20—C21	1.347 (9)	C54—C55	1.350 (8)
C20—H20A	0.9300	C54—H54A	0.9300
C21—C22	1.376 (6)	C55—H55A	0.9300
C21—H21A	0.9300	C56—C57	1.406 (18)
C22—H22A	0.9300	C56—H56A	0.9600
C23—N2	1.321 (4)	C56—H56B	0.9600
C23—C24	1.416 (5)	C56—H56C	0.9600
C23—C32	1.505 (5)	C57—C62	1.277 (18)
C24—C25	1.360 (5)	C57—C58	1.280 (18)
C24—H24A	0.9300	C58—C59	1.381 (13)
C25—O2	1.299 (4)	C58—H58A	0.9300
C25—C26	1.484 (5)	C59—C60	1.337 (14)
C26—C31	1.381 (5)	C59—H59A	0.9300
C26—C27	1.392 (5)	C60—C61	1.423 (15)
C27—C28	1.382 (6)	C60—H60A	0.9300
C27—H27A	0.9300	C61—C62	1.375 (15)
C28—C29	1.375 (8)	C61—H61A	0.9300
C28—H28A	0.9300	C62—H62A	0.9300
C29—C30	1.373 (8)		
			
O2—Cr1—N2	90.36 (10)	C31—C30—H30A	119.9
O2—Cr1—O1	93.05 (8)	C26—C31—C30	120.1 (5)
N2—Cr1—O1	170.66 (9)	C26—C31—H31A	119.9
O2—Cr1—Cl3	94.83 (7)	C30—C31—H31A	119.9
N2—Cr1—Cl3	91.88 (8)	C23—C32—H32A	109.5
O1—Cr1—Cl3	96.49 (7)	C23—C32—H32B	109.5
O2—Cr1—Cl1	168.99 (7)	H32A—C32—H32B	109.5
N2—Cr1—Cl1	96.76 (8)	C23—C32—H32C	109.5
O1—Cr1—Cl1	78.67 (6)	H32A—C32—H32C	109.5
Cl3—Cr1—Cl1	93.32 (3)	H32B—C32—H32C	109.5
O2—Cr1—Cl2	87.07 (7)	C34—C33—C38	119.9 (3)
N2—Cr1—Cl2	92.68 (8)	C34—C33—N3	120.4 (3)
O1—Cr1—Cl2	78.83 (6)	C38—C33—N3	119.5 (3)
Cl3—Cr1—Cl2	175.05 (3)	C33—C34—C35	119.2 (4)
Cl1—Cr1—Cl2	84.22 (3)	C33—C34—H34A	120.4
O3—Cr2—O1	169.72 (9)	C35—C34—H34A	120.4
O3—Cr2—N3	88.80 (10)	C36—C35—C34	120.1 (4)
O1—Cr2—N3	99.22 (10)	C36—C35—H35A	119.9
O3—Cr2—N1	93.04 (10)	C34—C35—H35A	119.9
O1—Cr2—N1	92.94 (9)	C37—C36—C35	121.1 (4)
N3—Cr2—N1	93.02 (11)	C37—C36—H36A	119.4
O3—Cr2—Cl2	91.68 (7)	C35—C36—H36A	119.4
O1—Cr2—Cl2	81.88 (6)	C36—C37—C38	119.4 (5)
N3—Cr2—Cl2	90.66 (8)	C36—C37—H37A	120.3
N1—Cr2—Cl2	174.07 (7)	C38—C37—H37A	120.3
O3—Cr2—Cl1	90.21 (7)	C33—C38—C37	120.1 (4)
O1—Cr2—Cl1	81.26 (6)	C33—C38—H38A	119.9
N3—Cr2—Cl1	175.20 (8)	C37—C38—H38A	119.9
N1—Cr2—Cl1	91.73 (8)	N3—C39—C40	123.1 (3)
Cl2—Cr2—Cl1	84.67 (3)	N3—C39—C48	119.8 (3)
Cr2—Cl1—Cr1	76.77 (3)	C40—C39—C48	117.0 (3)
Cr2—Cl2—Cr1	76.99 (3)	C41—C40—C39	125.4 (3)
C2—C1—C6	118.9 (3)	C41—C40—H40A	117.3
C2—C1—N1	120.9 (3)	C39—C40—H40A	117.3
C6—C1—N1	120.1 (3)	O3—C41—C40	123.0 (3)
C1—C2—C3	119.9 (4)	O3—C41—C42	113.3 (3)
C1—C2—H2A	120.0	C40—C41—C42	123.8 (3)
C3—C2—H2A	120.0	C43—C42—C47	118.3 (3)
C4—C3—C2	121.2 (4)	C43—C42—C41	118.6 (3)
C4—C3—H3A	119.4	C47—C42—C41	123.1 (3)
C2—C3—H3A	119.4	C42—C43—C44	121.3 (4)
C3—C4—C5	119.4 (4)	C42—C43—H43A	119.3
C3—C4—H4A	120.3	C44—C43—H43A	119.3
C5—C4—H4A	120.3	C45—C44—C43	119.8 (5)
C4—C5—C6	120.4 (4)	C45—C44—H44A	120.1
C4—C5—H5A	119.8	C43—C44—H44A	120.1
C6—C5—H5A	119.8	C46—C45—C44	120.1 (4)
C1—C6—C5	120.1 (4)	C46—C45—H45A	119.9
C1—C6—H6A	120.0	C44—C45—H45A	119.9
C5—C6—H6A	120.0	C45—C46—C47	120.6 (4)
N1—C7—C8	123.7 (3)	C45—C46—H46A	119.7
N1—C7—C16	121.7 (3)	C47—C46—H46A	119.7
C8—C7—C16	114.6 (3)	C46—C47—C42	119.7 (4)
C9—C8—C7	129.8 (3)	C46—C47—H47A	120.2
C9—C8—H8A	115.1	C42—C47—H47A	120.2
C7—C8—H8A	115.1	C39—C48—H48A	109.5
O1—C9—C8	123.3 (3)	C39—C48—H48B	109.5
O1—C9—C10	116.9 (2)	H48A—C48—H48B	109.5
C8—C9—C10	119.8 (3)	C39—C48—H48C	109.5
C15—C10—C11	118.3 (3)	H48A—C48—H48C	109.5
C15—C10—C9	121.4 (3)	H48B—C48—H48C	109.5
C11—C10—C9	120.4 (3)	C50—C49—H49A	109.5
C12—C11—C10	120.5 (3)	C50—C49—H49B	109.5
C12—C11—H11A	119.8	H49A—C49—H49B	109.5
C10—C11—H11A	119.8	C50—C49—H49C	109.5
C13—C12—C11	120.4 (3)	H49A—C49—H49C	109.5
C13—C12—H12A	119.8	H49B—C49—H49C	109.5
C11—C12—H12A	119.8	C51—C50—C55	118.4 (6)
C12—C13—C14	119.8 (3)	C51—C50—C49	120.8 (7)
C12—C13—H13A	120.1	C55—C50—C49	120.8 (7)
C14—C13—H13A	120.1	C50—C51—C52	121.1 (6)
C15—C14—C13	120.0 (3)	C50—C51—H51A	119.5
C15—C14—H14A	120.0	C52—C51—H51A	119.5
C13—C14—H14A	120.0	C53—C52—C51	120.3 (7)
C14—C15—C10	121.1 (3)	C53—C52—H52A	119.9
C14—C15—H15A	119.4	C51—C52—H52A	119.9
C10—C15—H15A	119.4	C52—C53—C54	118.4 (7)
C7—C16—H16A	109.5	C52—C53—H53A	120.8
C7—C16—H16B	109.5	C54—C53—H53A	120.8
H16A—C16—H16B	109.5	C55—C54—C53	121.0 (6)
C7—C16—H16C	109.5	C55—C54—H54A	119.5
H16A—C16—H16C	109.5	C53—C54—H54A	119.5
H16B—C16—H16C	109.5	C54—C55—C50	120.8 (6)
C18—C17—C22	120.5 (4)	C54—C55—H55A	119.6
C18—C17—N2	120.0 (4)	C50—C55—H55A	119.6
C22—C17—N2	119.5 (3)	C57—C56—H56A	109.5
C17—C18—C19	118.7 (5)	C57—C56—H56B	109.5
C17—C18—H18A	120.6	H56A—C56—H56B	109.5
C19—C18—H18A	120.6	C57—C56—H56C	109.5
C20—C19—C18	119.2 (6)	H56A—C56—H56C	109.5
C20—C19—H19A	120.4	H56B—C56—H56C	109.5
C18—C19—H19A	120.4	C62—C57—C58	124.8 (19)
C21—C20—C19	122.4 (6)	C62—C57—C56	112.8 (19)
C21—C20—H20A	118.8	C58—C57—C56	121.9 (18)
C19—C20—H20A	118.8	C57—C58—C59	118.1 (14)
C20—C21—C22	118.7 (6)	C57—C58—H58A	120.9
C20—C21—H21A	120.6	C59—C58—H58A	120.9
C22—C21—H21A	120.6	C60—C59—C58	127.1 (13)
C21—C22—C17	120.4 (5)	C60—C59—H59A	116.5
C21—C22—H22A	119.8	C58—C59—H59A	116.5
C17—C22—H22A	119.8	C59—C60—C61	106.0 (13)
N2—C23—C24	123.1 (3)	C59—C60—H60A	127.0
N2—C23—C32	120.3 (3)	C61—C60—H60A	127.0
C24—C23—C32	116.5 (3)	C62—C61—C60	128.9 (15)
C25—C24—C23	125.6 (3)	C62—C61—H61A	115.5
C25—C24—H24A	117.2	C60—C61—H61A	115.5
C23—C24—H24A	117.2	C57—C62—C61	114.2 (17)
O2—C25—C24	124.0 (3)	C57—C62—H62A	122.9
O2—C25—C26	114.6 (3)	C61—C62—H62A	122.9
C24—C25—C26	121.3 (3)	C7—N1—C1	119.3 (2)
C31—C26—C27	119.3 (4)	C7—N1—Cr2	124.1 (2)
C31—C26—C25	120.1 (3)	C1—N1—Cr2	116.61 (18)
C27—C26—C25	120.6 (3)	C23—N2—C17	117.3 (3)
C28—C27—C26	120.4 (5)	C23—N2—Cr1	123.6 (2)
C28—C27—H27A	119.8	C17—N2—Cr1	118.95 (19)
C26—C27—H27A	119.8	C39—N3—C33	120.6 (3)
C29—C28—C27	119.6 (5)	C39—N3—Cr2	122.8 (2)
C29—C28—H28A	120.2	C33—N3—Cr2	116.5 (2)
C27—C28—H28A	120.2	C9—O1—Cr2	124.71 (18)
C30—C29—C28	120.5 (5)	C9—O1—Cr1	131.18 (18)
C30—C29—H29A	119.7	Cr2—O1—Cr1	92.51 (8)
C28—C29—H29A	119.7	C25—O2—Cr1	127.0 (2)
C29—C30—C31	120.1 (5)	C41—O3—Cr2	126.5 (2)
C29—C30—H30A	119.9		
			
O3—Cr2—Cl1—Cr1	135.75 (7)	C62—C57—C58—C59	−4(2)
O1—Cr2—Cl1—Cr1	−38.50 (6)	C56—C57—C58—C59	−174.9 (13)
N3—Cr2—Cl1—Cr1	57.7 (9)	C57—C58—C59—C60	−6(2)
N1—Cr2—Cl1—Cr1	−131.20 (7)	C58—C59—C60—C61	9.1 (19)
Cl2—Cr2—Cl1—Cr1	44.08 (3)	C59—C60—C61—C62	−4(2)
O2—Cr1—Cl1—Cr2	−5.8 (4)	C58—C57—C62—C61	8(3)
N2—Cr1—Cl1—Cr2	−135.71 (8)	C56—C57—C62—C61	179.7 (12)
O1—Cr1—Cl1—Cr2	36.05 (6)	C60—C61—C62—C57	−4(2)
Cl3—Cr1—Cl1—Cr2	132.00 (4)	C8—C7—N1—C1	176.5 (3)
Cl2—Cr1—Cl1—Cr2	−43.69 (3)	C16—C7—N1—C1	−4.2 (5)
O3—Cr2—Cl2—Cr1	−134.02 (7)	C8—C7—N1—Cr2	−3.1 (4)
O1—Cr2—Cl2—Cr1	37.94 (6)	C16—C7—N1—Cr2	176.3 (3)
N3—Cr2—Cl2—Cr1	137.16 (8)	C2—C1—N1—C7	92.6 (4)
N1—Cr2—Cl2—Cr1	8.7 (8)	C6—C1—N1—C7	−90.6 (4)
Cl1—Cr2—Cl2—Cr1	−43.97 (3)	C2—C1—N1—Cr2	−87.8 (3)
O2—Cr1—Cl2—Cr2	−129.28 (7)	C6—C1—N1—Cr2	89.0 (3)
N2—Cr1—Cl2—Cr2	140.50 (8)	O3—Cr2—N1—C7	−178.3 (3)
O1—Cr1—Cl2—Cr2	−35.58 (6)	O1—Cr2—N1—C7	10.1 (3)
Cl3—Cr1—Cl2—Cr2	−16.5 (4)	N3—Cr2—N1—C7	−89.4 (3)
Cl1—Cr1—Cl2—Cr2	43.97 (3)	Cl2—Cr2—N1—C7	39.0 (9)
C6—C1—C2—C3	2.8 (6)	Cl1—Cr2—N1—C7	91.4 (2)
N1—C1—C2—C3	179.6 (4)	O3—Cr2—N1—C1	2.1 (2)
C1—C2—C3—C4	−1.4 (7)	O1—Cr2—N1—C1	−169.5 (2)
C2—C3—C4—C5	−0.4 (7)	N3—Cr2—N1—C1	91.1 (2)
C3—C4—C5—C6	0.7 (7)	Cl2—Cr2—N1—C1	−140.6 (7)
C2—C1—C6—C5	−2.5 (6)	Cl1—Cr2—N1—C1	−88.2 (2)
N1—C1—C6—C5	−179.3 (3)	C24—C23—N2—C17	177.2 (3)
C4—C5—C6—C1	0.8 (6)	C32—C23—N2—C17	−1.2 (5)
N1—C7—C8—C9	−7.6 (6)	C24—C23—N2—Cr1	−7.1 (5)
C16—C7—C8—C9	173.1 (3)	C32—C23—N2—Cr1	174.5 (3)
C7—C8—C9—O1	5.6 (5)	C18—C17—N2—C23	−93.7 (4)
C7—C8—C9—C10	−176.5 (3)	C22—C17—N2—C23	86.4 (4)
O1—C9—C10—C15	29.8 (4)	C18—C17—N2—Cr1	90.5 (4)
C8—C9—C10—C15	−148.3 (3)	C22—C17—N2—Cr1	−89.5 (3)
O1—C9—C10—C11	−151.6 (3)	O2—Cr1—N2—C23	20.3 (3)
C8—C9—C10—C11	30.3 (4)	O1—Cr1—N2—C23	131.8 (5)
C15—C10—C11—C12	0.4 (5)	Cl3—Cr1—N2—C23	−74.6 (3)
C9—C10—C11—C12	−178.3 (3)	Cl1—Cr1—N2—C23	−168.2 (3)
C10—C11—C12—C13	−0.3 (5)	Cl2—Cr1—N2—C23	107.4 (3)
C11—C12—C13—C14	−0.2 (6)	O2—Cr1—N2—C17	−164.2 (2)
C12—C13—C14—C15	0.5 (6)	O1—Cr1—N2—C17	−52.6 (7)
C13—C14—C15—C10	−0.4 (5)	Cl3—Cr1—N2—C17	101.0 (2)
C11—C10—C15—C14	0.0 (5)	Cl1—Cr1—N2—C17	7.4 (2)
C9—C10—C15—C14	178.6 (3)	Cl2—Cr1—N2—C17	−77.1 (2)
C22—C17—C18—C19	−0.8 (6)	C40—C39—N3—C33	−171.2 (3)
N2—C17—C18—C19	179.2 (4)	C48—C39—N3—C33	10.0 (5)
C17—C18—C19—C20	0.5 (8)	C40—C39—N3—Cr2	12.6 (5)
C18—C19—C20—C21	−1.0 (9)	C48—C39—N3—Cr2	−166.2 (3)
C19—C20—C21—C22	1.7 (9)	C34—C33—N3—C39	−100.1 (4)
C20—C21—C22—C17	−2.0 (7)	C38—C33—N3—C39	84.7 (4)
C18—C17—C22—C21	1.6 (6)	C34—C33—N3—Cr2	76.3 (4)
N2—C17—C22—C21	−178.5 (4)	C38—C33—N3—Cr2	−98.9 (3)
N2—C23—C24—C25	−9.6 (6)	O3—Cr2—N3—C39	−28.1 (3)
C32—C23—C24—C25	168.9 (4)	O1—Cr2—N3—C39	145.4 (2)
C23—C24—C25—O2	3.7 (6)	N1—Cr2—N3—C39	−121.1 (3)
C23—C24—C25—C26	−172.9 (4)	Cl2—Cr2—N3—C39	63.6 (3)
O2—C25—C26—C31	−39.0 (5)	Cl1—Cr2—N3—C39	50.0 (11)
C24—C25—C26—C31	137.9 (4)	O3—Cr2—N3—C33	155.5 (2)
O2—C25—C26—C27	140.9 (3)	O1—Cr2—N3—C33	−30.9 (2)
C24—C25—C26—C27	−42.2 (5)	N1—Cr2—N3—C33	62.6 (2)
C31—C26—C27—C28	0.6 (6)	Cl2—Cr2—N3—C33	−112.8 (2)
C25—C26—C27—C28	−179.3 (4)	Cl1—Cr2—N3—C33	−126.3 (9)
C26—C27—C28—C29	−0.8 (7)	C8—C9—O1—Cr2	6.5 (4)
C27—C28—C29—C30	0.0 (8)	C10—C9—O1—Cr2	−171.47 (18)
C28—C29—C30—C31	1.0 (8)	C8—C9—O1—Cr1	−126.1 (3)
C27—C26—C31—C30	0.4 (6)	C10—C9—O1—Cr1	55.9 (3)
C25—C26—C31—C30	−179.7 (4)	O3—Cr2—O1—C9	−137.3 (5)
C29—C30—C31—C26	−1.2 (7)	N3—Cr2—O1—C9	81.7 (2)
C38—C33—C34—C35	2.5 (5)	N1—Cr2—O1—C9	−11.8 (2)
N3—C33—C34—C35	−172.7 (3)	Cl2—Cr2—O1—C9	171.0 (2)
C33—C34—C35—C36	−0.6 (6)	Cl1—Cr2—O1—C9	−103.1 (2)
C34—C35—C36—C37	−1.2 (7)	O3—Cr2—O1—Cr1	9.0 (5)
C35—C36—C37—C38	1.1 (8)	N3—Cr2—O1—Cr1	−131.98 (9)
C34—C33—C38—C37	−2.6 (6)	N1—Cr2—O1—Cr1	134.48 (9)
N3—C33—C38—C37	172.6 (4)	Cl2—Cr2—O1—Cr1	−42.63 (6)
C36—C37—C38—C33	0.8 (7)	Cl1—Cr2—O1—Cr1	43.18 (6)
N3—C39—C40—C41	10.4 (6)	O2—Cr1—O1—C9	−88.2 (2)
C48—C39—C40—C41	−170.7 (3)	N2—Cr1—O1—C9	160.5 (5)
C39—C40—C41—O3	−5.9 (5)	Cl3—Cr1—O1—C9	7.0 (2)
C39—C40—C41—C42	173.6 (3)	Cl1—Cr1—O1—C9	99.1 (2)
O3—C41—C42—C43	−15.5 (5)	Cl2—Cr1—O1—C9	−174.6 (2)
C40—C41—C42—C43	165.0 (4)	O2—Cr1—O1—Cr2	129.10 (9)
O3—C41—C42—C47	166.7 (3)	N2—Cr1—O1—Cr2	17.8 (6)
C40—C41—C42—C47	−12.8 (5)	Cl3—Cr1—O1—Cr2	−135.68 (6)
C47—C42—C43—C44	−2.6 (7)	Cl1—Cr1—O1—Cr2	−43.58 (6)
C41—C42—C43—C44	179.5 (5)	Cl2—Cr1—O1—Cr2	42.69 (6)
C42—C43—C44—C45	−0.1 (9)	C24—C25—O2—Cr1	20.0 (5)
C43—C44—C45—C46	3.0 (10)	C26—C25—O2—Cr1	−163.2 (2)
C44—C45—C46—C47	−3.2 (9)	N2—Cr1—O2—C25	−27.0 (3)
C45—C46—C47—C42	0.5 (8)	O1—Cr1—O2—C25	161.7 (3)
C43—C42—C47—C46	2.3 (6)	Cl3—Cr1—O2—C25	64.9 (3)
C41—C42—C47—C46	−179.9 (4)	Cl1—Cr1—O2—C25	−157.4 (3)
C55—C50—C51—C52	3.0 (9)	Cl2—Cr1—O2—C25	−119.7 (3)
C49—C50—C51—C52	−178.9 (7)	C40—C41—O3—Cr2	−23.6 (4)
C50—C51—C52—C53	−2.5 (11)	C42—C41—O3—Cr2	156.8 (2)
C51—C52—C53—C54	0.6 (11)	O1—Cr2—O3—C41	−107.4 (5)
C52—C53—C54—C55	0.6 (10)	N3—Cr2—O3—C41	34.2 (2)
C53—C54—C55—C50	−0.1 (9)	N1—Cr2—O3—C41	127.1 (2)
C51—C50—C55—C54	−1.8 (9)	Cl2—Cr2—O3—C41	−56.5 (2)
C49—C50—C55—C54	−179.9 (6)	Cl1—Cr2—O3—C41	−141.1 (2)
